# Importance of spliceosomal RNP1 motif for intermolecular T-B cell spreading and tolerance restoration in lupus

**DOI:** 10.1186/ar2317

**Published:** 2007-10-26

**Authors:** Fanny Monneaux, Véronique Parietti, Jean-Paul Briand, Sylviane Muller

**Affiliations:** 1 Centre National de la Recherche Scientifique UPR9021, Institut de Biologie Moléculaire et Cellulaire, 15 rue René Descartes, 67000 Strasbourg, France

## Abstract

We previously demonstrated the importance of the RNP1 motif-bearing region 131–151 of the U1-70K spliceosomal protein in the intramolecular T-B spreading that occurs in MRL/lpr lupus mice. Here, we analyze the involvement of RNP1 motif in the development and prevention of naturally-occurring intermolecular T-B cell diversification. We found that MRL/lpr peripheral blood lymphocytes proliferated in response to peptides containing or corresponding exactly to the RNP1 motif of spliceosomal U1-70K, U1-A and hnRNP-A2 proteins. We also demonstrated that rabbit antibodies to peptide 131–151 cross-reacted with U1-70K, U1-A and hnRNP-A2 RNP1-peptides. These antibodies recognized the U1-70K and U1-A proteins, and also U1-C and SmD1 proteins, which are devoid of RNP1 motif. Repeated administration of phosphorylated peptide P140 into MRL/lpr mice abolished T-cell response to several peptides from the U1-70K, U1-A and SmD1 proteins without affecting antibody and T-cell responses to foreign (viral) antigen in treated mice challenged with infectious virus. These results emphasized the importance of the dominant RNP1 region, which seems to be central in the activation cascade of B and T cells reacting with spliceosomal RNP1^+ ^and RNP1^- ^spliceosomal proteins. The tolerogenic peptide P140, which is recognized by lupus patients' CD4^+ ^T cells and known to protect MRL/lpr mice, is able to thwart emergence of intermolecular T-cell spreading in treated animals.

## Introduction

Longitudinal studies of spontaneously lupus-prone inbred mouse strains and patients with systemic lupus erythematosus (SLE) consistently show an ordered appearance of typical auto-antibodies in the serum of individuals [1-4]. With time the fine specificity of the antibody response initially focused against one or few autoepitopes diversifies to other epitopes of the same protein (intramolecular spreading) and to other components that are physically associated within the same antigenic macromolecular particles, such as nucleosome, spliceosome, and Ro particle (intermolecular spreading). Epitope spreading is thus a process whereby epitopes distinct from and non-cross-reactive with an inducing epitope become major targets of an ongoing immune response. This phenomenon is not limited to autoimmunity; it has also been described in experimental and natural situations as a consequence of acute or persistent infection. Although the concept of epitope spreading was introduced more than 15 years ago [5], the cellular components that catalyze the spreading hierarchy have not been well defined, and certain aspects of this process remain unexplained. Recent studies suggest that autoreactive B cells are important cellular mediators contributing to autoreactive T-cell response diversification via their functions that mediate antigen processing and presentation [6,7].

Previous work from our laboratory demonstrated that peptide 131–151 of the spliceosomal U1-70K protein as well as a peptide analogue containing a phosphoserine residue at position 140 (peptide P140) are recognized by CD4^+ ^T cells from lupus mice. Both peptides were shown to behave as promiscuous epitopes and bind a large panel of murine and human MHC class II molecules [8-11]. Administration into young MRL/lpr lupus-prone mice of P140 peptide in saline, but not of the non-phosphorylated peptide 131–151, led to a dramatic amelioration of the clinical and biological manifestations of treated animals and significantly prolonged their survival [9]. The peptide P140 administrated in Freund's adjuvant (FA) accelerated the renal disease in MRL/lpr mice [9]. Our studies revealed further that peripheral CD4^+ ^T cells from lupus patients, but not from patients with other autoimmune diseases (such as rheumatoid arthritis, primary Sjögren's syndrome, autoimmune deafness, polymyositis, primary billiary cirrhosis and autoimmune hepatitis) or infectious diseases, very specifically recognized the 131–151 and P140 peptides, and that phosphorylation of Ser^140 ^prevented *ex vivo *proliferation of lupus patients' CD4^+ ^T cells but not secretion of high levels of regulatory cytokines [11].

The 131–151 sequence of the spliceosomal U1-70K protein is located within a 80–90 amino acid-long RNA-binding domain. It encompasses a conserved sequence, called RNP1 motif, which is also present in other RNA-binding proteins, such as small nuclear (sn)RNP (such as U1-A) and heterogeneous nuclear (hn)RNP (such as hnRNP-A2/B1) proteins. Starting from the observation that sequences containing this RNP1 motif are often targeted by antibodies from lupus patients and mice, we hypothesized that the RNP1 motif could be involved in the earliest stages of the T-B intramolecular diversification process to other regions of one of the spliceosomal proteins that contain this unique motif, and might promote intermolecular spreading to epitopes of other proteins present within the same spliceosomal particle and containing or not an RNP1 motif [12,13]. We demonstrated that an intramolecular T and B cell spreading effectively occurs in MRL/lpr mice tested at different ages and emphasized the importance of the RNP1 region in the cascade of events observed in the murine lupus response [14]. In the present study, we investigated the potential capacity of peptide 131–151 to generate antibodies that react with spliceosomal peptides and proteins encompassing, or not, the RNP1 motif. We tested the reactivity of peripheral blood lymphocytes (PBLs) from unprimed lupus mice to react with RNP1-containing peptides from several spliceosomal proteins. We also examined whether administration of protecting P140 peptide into MRL/lpr mice could modulate intermolecular epitope spreading *in vivo *by preventing T-cell response to peptides from heterologous spliceosomal proteins that do or do not contain an RNP1 motif. We provide data showing that P140 peptide treatment efficiently suppresses T-cell responses to other, unrelated spliceosomal epitopes, in addition to the tolerizing peptide. Remarkably, however, the B and T cell immune response to foreign antigens (such as viral particles) remains unaffected in treated animals.

## Materials and methods

### Peptides and proteins

**Figure 1:**
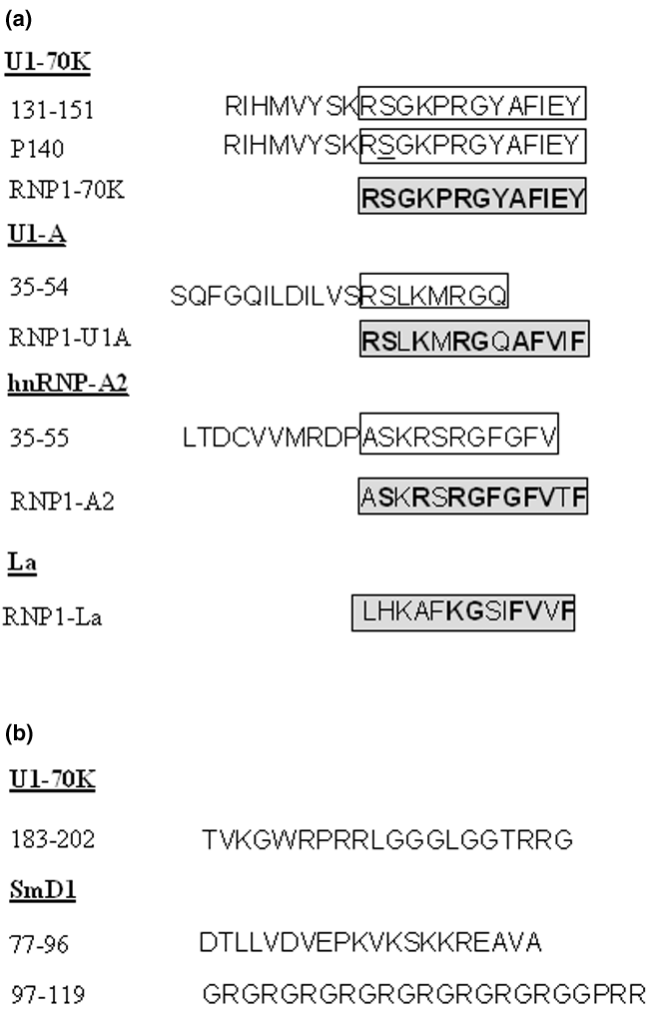
Synthetic peptides used in this study.** (a)** Peptides of U1-A, U1-70K, hnRNP-A2 and La proteins containing or corresponding to RNP1 motifs. The RNP1 motif (complete or partial) is highlighted by an empty box. In peptides corresponding exactly to the motif (gray boxes) amino acid similarities are in bold face. The serine^140 ^residue, which is phosphorylated in the P140 peptide is underlined. **(b)** Sequence of other peptides used in this study.

Eleven synthetic peptides of the U1-70K, U1-A, SmD1, hnRNP-A2 and La ribonucleoproteins were used in this study (Figure 1). Six of them were described previously, namely 131–151, 183–202 and P140 peptides of the U1-70K protein, peptide 35–55 of the hnRNP-A2 protein, and peptides 77–96 and 97–119 of SmD1 [3,8,15,16]. The synthesis of new peptides, including four peptides, the sequence of which corresponded exactly to the respective RNP1 motifs of the four proteins U1-70K, U1-A, hnRNP-A2 and La, and peptide 35–54 of the U1-A protein was performed using the same classical N-[9-fluorenyl] methoxycarbonyl (Fmoc) solid-phase chemistry. The homogeneity of each purified peptide was checked by analytical high-performance liquid chromatography (HPLC), and their identity was assessed by matrix-assisted laser desorption and ionization time-of-flight (TOF) mass spectrometry (MS) using a Protein TOF apparatus (Bruker Spectrospin, Bremen, Germany). A large fragment encompassing residues 21–119 of the SmD1 protein (human sequence [15]) was assembled using optimized Fmoc chemistry protocols with a multichannel peptide synthesizer [17]. Ser^35^, Ser^59 ^and Thr^78 ^residues were incorporated into the growing peptide chain by pseudoproline-protected dipeptides to increase solvation and coupling rates during peptide assembly [18]. Side chain deprotection and cleavage of peptides from the solid support was performed by trifluoroacetic acid (TFA) treatment in the presence of appropriate scavengers. The SmD1 fragment 21–119 was purified by reversed-phase HPLC (RP-HPLC) using a Beckman preparative HPLC system on a Nucleosil C18 (1 × 30 cm) column. The elution was achieved with a linear gradient of aqueous 0.1% TFA (A) and 0.08% TFA in 80% acetonitrile, 20% water (B) at a flow rate of 6 ml/min with UV detection at 230 nm. The purity of the SmD1 fragment was controlled by analytical RP-HPLC on a Beckman instrument on two types of column, namely a Nucleosil C18 5 μm-column (150 × 4.6 mm) and a Nucleosil C4 5 μm-column (150 × 4.6 mm), using a linear gradient of 0.1% TFA in water and acetonitrile containing 0.08% TFA at a flow rate of 1.2 ml/min. The mass of the long SmD1 fragment 21–119 was assessed by LC/MS using a ThermoFinnigan LCQ advantage/LC surveyor. Recombinant U1-A, U1-C, U1-70K and SmBB' proteins (human sequences) were purchased from Diarect AG (Freiburg, Germany).

### Rabbit immunization

On days 1, 21 and 42, outbred Fauves de Bourgogne female rabbits (Dombes Romans, Chatillon, France) were injected subcutaneously (sc) with peptide 131–151 of the U1-70K protein (100 μg/rabbit/injection) emulsified in complete FA (CFA) for the first injection and incomplete FA for the subsequent injections. Rabbits were bled before immunization and then regularly throughout the protocol.

### ELISA and Western immunoblotting

Standard ELISA procedures with peptide 131–151 (2 μM in 0.05 M carbonate buffer, pH 9.6) or dsDNA (100 ng/ml in 25 mM citrate buffer, pH 5.4) directly coated onto polyvinyl microtiter plates (Falcon, Oxnard, California, USA), and goat anti-rabbit IgG-horseradish peroxidase (HRP) conjugate (Jackson ImmunoResearch Laboratories, West Groves, Pennsylvania) diluted 1:50,000 in phosphate-buffered saline containing 0.05% Tween (PBS-T) were used to measure antibody reactivity in rabbit antisera [8]. The final reaction was visualized with H2O2 and 3,3',5,5'-tetramethyl benzidine used as chromogen, and absorbance was measured at 450 nm. For competitive ELISA, increasing amounts of inhibitor peptides were first incubated for 1 h at 37°C and then overnight at 4°C with constant dilutions of rabbit antisera in PBS-T. The mixtures were subsequently transferred to polyvinyl Falcon plates pre-coated with peptide 131–151, and post-coated with bovine serum albumin in PBS-T (0.4% w/v). After a 1 h incubation at 37°C and washing, bound antibodies were detected as indicated above. Preliminary experiments were conducted in a direct format to define the working dilution of rabbit antiserum. For Western immunoblotting, U1-A, U1-C, U1-70K, SmBB' and SmD1 proteins were first subjected to electrophoresis in 12.5% SDS-polyacrylamide gels and then transferred to nitrocellulose. The blotted strips were saturated in Tris-buffered saline, pH 7.5, containing 0.5% Tween (TBS-T) and 5% milk for 1 h at room temperature (RT), and then incubated with rabbit antisera diluted 1:500 in TBS-T-milk for 1 h at RT. After washing, strips were incubated with HRP-conjugated second antibodies to rabbit IgG (1:5,000 in TBS-T). Enhanced chemiluminescent (ECL™) reagents (Amersham Pharmacia Biotech, Buckinghamshire, UK) were used to reveal positive reactions.

### Cellular assays

To study the natural T-cell reactivity occurring in unprimed lupus-prone mice, MRL/lpr mice were bled at weeks 8, 10, 12 and 14, and lymphocytes were purified by density separation (Lympholyte-M, d = 1.0875; Cedarlane, Hornby, Canada). PBLs were collected, washed three times, and resuspended at 3 × 10^6 ^cells/ml in L-alanyl-L-glutamine-enriched RPMI 1640 medium (Cambrex, Verviers, Belgium) containing 10% fetal calf serum (FCS; Dutscher, Brumath, France), HEPES, gentamycine, and β-mercaptoethanol. The proliferative response to peptides was measured in duplicate using 3 × 10^5 ^cells/well and a single peptide concentration (100 μM). After 72 h, the cultures were pulsed for 18 h with [^3^H]-thymidine (6.7 Ci/mmol; 1μCi/well) and DNA-incorporated radioactivity was measured using a Matrix 9600 direct beta counter (Packard, Meriden, Connecticut, USA; cpm range from 100 to 30,000). The SD of duplicate cultures was always below 20% of the mean. Control tests were performed by adding Con-A (100 μl/well; 5 μg/ml) to cells during the time of the culture (90 h).

To analyze the effect of P140 peptide administration on spontaneous T cell spreading occurring in lupus-prone mice, MRL/lpr mice received peptide P140 (100 μg in saline/mouse/injection; 10 mice) or PBS alone (10 mice) intravenously (iv) at weeks 5, 7 and 9 [9]. Treated mice were bled at week 10, and peptides of interest (100 μM in the cultures) were tested for their ability to induce proliferation of PBLs, as described above.

To examine the possible effect of P140 treatment on the ability of mice to mount a normal immune reaction after viral infection, P140-treated MRL/lpr mice (18-week-old at the time of the experiment) were challenged intranasally (in) with A/NT/60/68 influenza virus (H3N2 strain) in allantoic fluid using a dose predetermined to provoke ~30% of weight loss [19]. The required dose was of the order of 40 μl of undiluted allantoic fluid (HA titer 1260) per mouse. As a control, a group of untreated MRL/lpr mice was challenged in parallel. Mice were followed daily for their body weight. The capacity of PBLs from challenged mice to proliferate and secrete INF-γ *ex vivo *in the presence of increasing concentrations of the HA peptide 307–319 (PKYVKQNTLKLAT [20]) representing a promiscuous T-helper epitope from influenza virus HA, was evaluated 13 days after challenge, as described above. The anti-virus antibody production in the serum from challenged mice was measured in ELISA [19] 28 days after challenge.

All animal experiments were performed with the approval of the local Institutional Animal Care and Use Committee (CREMEAS).

### Statistics

Analysis for statistically significant differences was performed with Student's *t*-test. *P *values < 0.05 were considered significant.

## Results

### Recognition by T cells from unprimed MRL/lpr mice of peptides containing or corresponding exactly to the RNP1 motif

**Figure 2:**
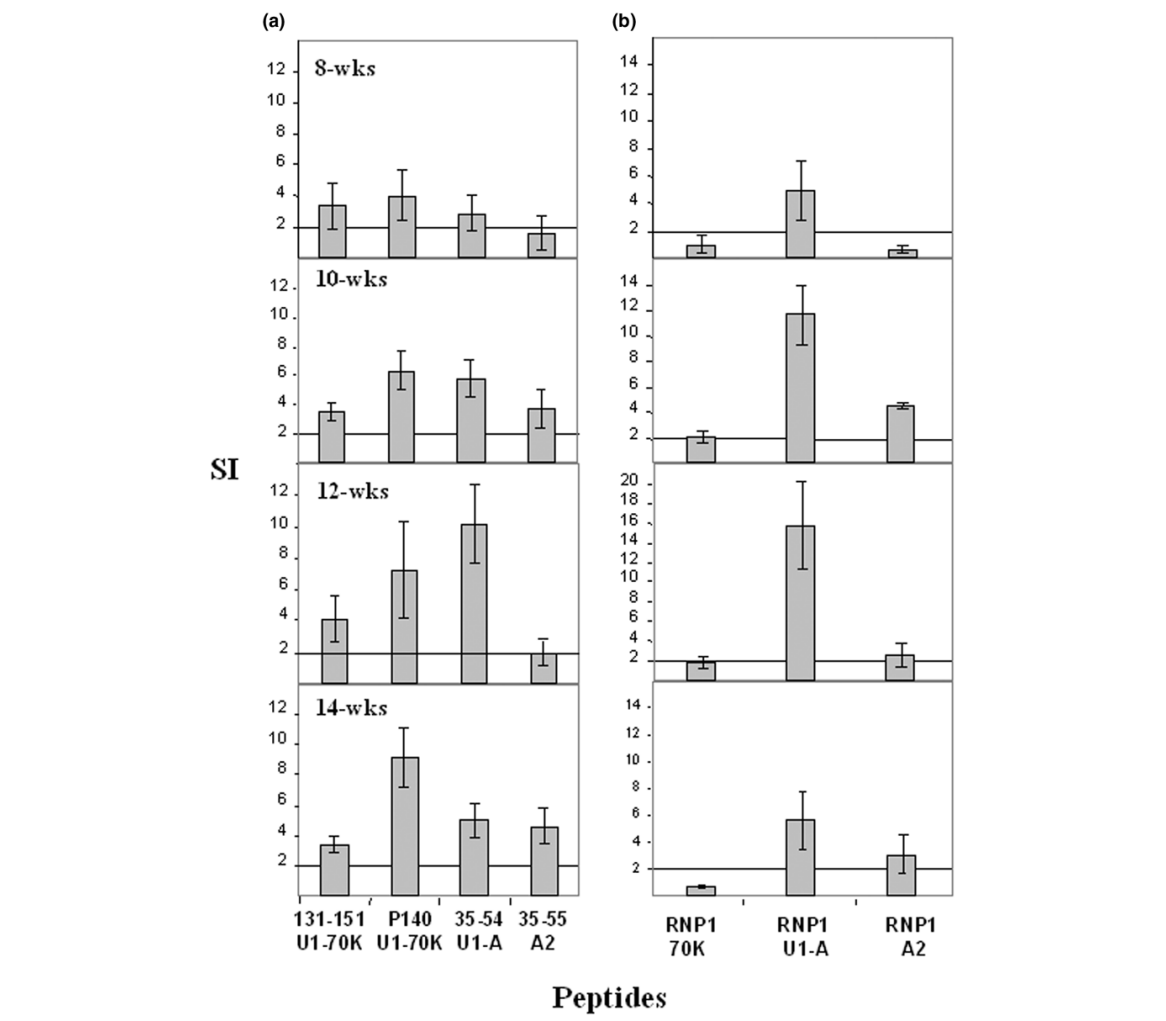
Spontaneous T-cell reactivity to peptides containing or corresponding to RNP1 motifs in unprimed MRL/lpr mice. The proliferative response of peripheral blood lymphocytes (PBLs) from 8-, 10-, 12- and 14-week-old mice was measured in the presence of U1-70K, U1-A and hnRNP-A2 peptides (100 μM) encompassing completely or partially the RNP1 motif of each protein **(a)** or corresponding exactly to the RNP1 motif of each protein **(b)**. The results are expressed as SI corresponding to the ratio cpm in the culture with peptide to cpm in the culture without peptide. A mean SI > 2 was considered to be positive (horizontal line). The average tritiated thymidine incorporation in the absence of peptide and in the presence of Con-A was 50 and 3,000 cpm, respectively. This experiment is one of two individual experiments that showed similar results. Bars show the mean ± SEM.

In a first set of experiments, 20 non-immunized MRL/lpr mice were bled longitudinally at weeks 8, 10, 12 and 14, and in order to have enough cells to test individually several peptides in duplicate (only 1.5 to 2.10^6 ^PBLs can be collected from the blood of a single living mouse), PBLs were pooled and tested for their ability to proliferate *ex vivo *in response to 100 μM of each peptide. Owing to the well-documented accumulation of CD4^-^CD8^- ^double negative (DN) T cells in PBLs of MRL/lpr mice (80% of DN T cells at 17 weeks of age, data not shown), we focused our measurement of specific CD4^+ ^T cells proliferation in a window ending at week 14. Responses were considered to be positive when stimulation indices (SI) were higher than 2 in the proliferation assay. In good agreement with previous findings [14], a proliferative response of PBLs purified from 8 to 14-week-old MRL/lpr mice was observed in response to peptides 131–151 and P140 of the U1-70K protein (Figure 2a). In addition, U1-A peptide 35–54, which contains a part of the RNP1 motif (Figure 1a), induced proliferation of MRL/lpr PBLs that was similar in intensity to the proliferative response to P140 peptide (Figure 2a), and was at its maximum at week 12. A proliferative response was also measured when PBLs were recalled with hnRNP-A2 peptide 35–55 (Figure 2a), which also contains only a part of the RNP1 motif (Figure 1a).

In order to determine whether MRL/lpr T cells precisely recognized the RNP1 motif present in the respective spliceosomal proteins, we synthesized three additional peptides corresponding exactly to the RNP1 motifs of the U1-70K, U1-A and hnRNP-A2 proteins, called RNP1-70K, RNP1-U1A and RNP1-A2, respectively (Figure 1a), and tested, as above, the ability of these peptides to stimulate the proliferation of PBLs from 8-14-wk-old MRL/lpr mice (Figure 2b). No or very weak proliferation was observed in response to the RNP1-70K peptide 139–151 at any age. A modest but significant proliferative response with RNP1-A2 peptide 45–57 was measurable with PBLs from 10, 12 and 14-wk-old MRL/lpr mice. In contrast, as early as 8 weeks of age, and at least until 14 weeks, a strong proliferative response was observed when MRL/lpr PBLs were cultured in the presence of the 13-residue-long RNP1-U1A peptide 47–59 (Figure 2b; maximum at week 12). This response was inhibited in the presence of neutralizing anti-CD4 monoclonal antibody GK1.5 (10 μg/ml; not shown) indicating that the main cell population reacting with the RNP1-U1A peptide 47–59 does correspond to CD4^+ ^T cells.

### Reactivity of rabbit antibodies to peptide 131–151 of the U1-70K protein with RNP1-peptide

**Figure 3:**
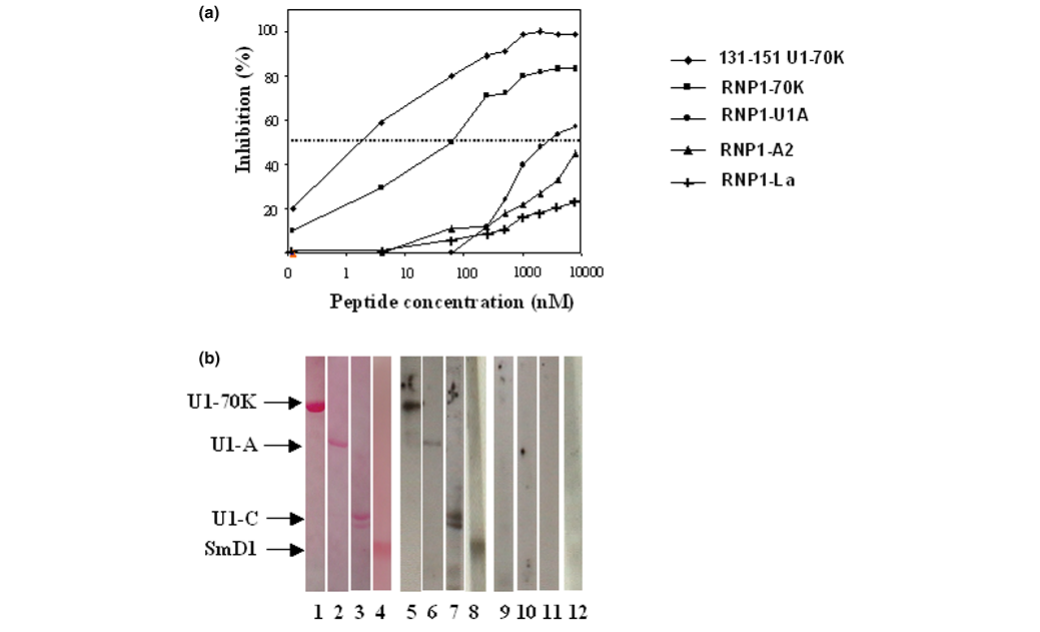
Reactivity of rabbit antibodies directed to peptide 131–151 of the U1-70K protein with RNP1-peptides and spliceosomal proteins. **(a) **The antiserum was diluted 1/20,000 and incubated first for 1 h at 37°C and then overnight at 4°C with increasing amounts of different RNP1-peptides used as competitors in the fluid-phase. The homologous peptide 131–151 was used as control. The mixtures were then added to microtiter plates pre-coated with 2 μM of immunizing peptide 131–151. The results are expressed as the percentage of inhibition of the ELISA reaction measured without competitor peptide. **(b)** U1-70K, U1-A, U1-C, and SmD1 proteins were subjected to electrophoresis, transferred to nitrocellulose, and colored with Ponceau red (lanes 1–4). The blotted strips were then incubated with the serum (diluted 1/500) from rabbits that received either peptide 131–151 (lanes 5–8) or CFA alone (lanes 9–12). IgG antibodies only were tested. ECL reagents were used to reveal positive reactions.

In our diversification model [13], the RNP1 motif could be involved at an early stage of the anti-spliceosomal autoimmune response. We first used competitive ELISA to measure the cross-capacity of antibodies generated against the RNP1 sequence of the U1-70K protein to recognize RNP1-peptides from U1-A and hnRNP-A2 proteins (Figure 3a). The binding to the plastic-bound 131–151 peptide of IgG antibodies from an outbred rabbit immunized against the same peptide 131–151 was almost completely inhibited by the homologous peptide 131–151, and to a lower extent by the RNP1-70K peptide 139–151. The amount of peptide required to inhibit 50% of the antibody reaction was 5 nM of the homologous peptide 131–151 and 80 nM of the RNP1-70K peptide. Although much more poorly (IC_50 _~3 μM), RNP1-U1A peptide was also able to inhibit the IgG antibody binding to peptide 131–151 in a dose-dependent manner. However, both the RNP1-A2 and RNP1-La peptides, the latter significantly differing in its sequence from the RNP1 sequence present in the U1-70K protein (Figure 1a), were very poor inhibitors (Figure 3a). These findings indicate that antibodies raised to peptide 131–151 of the U1-70K protein recognize the RNP1 motif present within U1-A, but also show that punctual mutations present in the respective RNP1 motifs (Figure 1a) strongly affect their conformation in solution and consequently their cross-reactivity with antibodies.

### Antibodies generated in outbred rabbits to peptide 131–151 of the U1-70K protein react with the cognate protein and with other spliceosomal proteins

We then questioned whether antibodies raised to peptide 131–151 are able to recognize the whole cognate protein and other proteins present in the same spliceosomal particle (Figure 3b). Ponceau red staining of membranes blotted with the three recombinant proteins U1-70K, U1-A, and U1-C and with the long synthetic fragment 21–119 of SmD1 revealed a single band at 65 kD, 35 kD, 20 kD and 12 kD, respectively, as determined with MW markers (Figure 3b, lanes 1–4). IgG antibodies from the rabbit immunized against peptide 131–151 in CFA (see above) strongly reacted with the U1-70K (lane 5) and the U1-A (lane 6) proteins, as well as with the U1-C (lane 7) and SmD1 (lane 8) proteins, which do not contain any RNP1 motif, suggesting the establishment of a heterologous B-cell epitope spreading phenomenon, and not solely immune cross-reactivity. No reactivity was found with SmBB' protein (not shown) or with any of the five proteins when tested with the serum from an outbred rabbit that received several injections of CFA alone (lanes 9–12). This rabbit did not develop any IgG reactivity to dsDNA (as measured by ELISA), and no clinical sign of autoimmunity was measurable 8 weeks after the last of three peptide administrations (result confirmed in several outbred rabbits). New Zealand White rabbits were not used in this set of experiments because they have a natural tendency to produce auto-antibodies [21]. A similar result was obtained with two outbred mice (ICR, Harlan) immunized with peptide 131–151, for which antisera react in Western immunoblot with the U1-70K protein and also with U1-A and U1-C proteins (data not shown).

### Effect of P140 therapy on spontaneous T cell spreading

**Figure 4:**
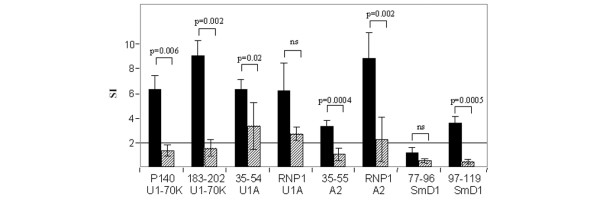
Effect of a brief peptide P140 therapy on the spontaneous T cell spreading in MRL/lpr mice. Mice were either treated with peptide P140 (hatched bars) administrated intravenously in saline at weeks 5, 7 and 9 or received saline alone (solid bars). The proliferative response of PBLs was measured *ex vivo *at 10 weeks in the presence of selected peptides (100 μM) containing (P140, 35–54 U1-A, RNP1-U1A, 35–55 hnRNP-A2, RNP1-A2) or not (183–202 of the U1-70K protein, 97–119 SmD1) an RNP1 motif. A mean SI > 2 was considered to be positive (horizontal line). The average tritiated thymidine incorporation in the absence of peptide and in the presence of Con-A was 50 and 2,000 cpm, respectively. Bars show the mean ± SEM. Significant differences are indicated. ns: non-significant reduction.

We previously demonstrated that phosphorylated P140 peptide administrated in saline into MRL/lpr mice transiently abolished T cell spreading to other regions of the cognate protein U1-70K [14]. We hypothesized that through its RNP1 motif, the P140 peptide could originate a mechanism of 'tolerance spreading' leading to the beneficial effect observed in treated MRL/lpr mice. With the aim of further investigating whether peptide P140 could also alter spontaneous intermolecular T cell spreading in this pathway, we studied the proliferative response of PBLs collected from P140-treated MRL/lpr mice to a set of selected spliceosomal peptides. We confirmed our previous findings showing that the proliferative response of PBLs from P140-treated mice to the homologous peptide P140 and to peptide 183–202 of the same parent U1-70K protein was significantly abolished (79 and 83% of inhibition, respectively; *P *= 0.006 and *P *= 0.002, respectively; Figure 4). In the same assay, a 56%-reduction of the proliferative response to the RNP1-UA peptide was detectable (Figure 4) but was not statistically significant (*P *= 0.059). However, we found a statistically significant drop of PBL proliferative response in the presence of peptide 35–54 of U1-A (47% decrease; *P *= 0.02), peptide 35–55 of hnRNP-A2 (67% decrease; *P *= 0.0004) and the RNP1 A2 peptide (74% decrease; *P *= 0.002).

As U1-A and hnRNP-A2 peptides do contain an RNP1 motif, we could not rule out the possibility that the observed drop of responsiveness was simply due to the extinction of P140-specific T cells by a homologous-like, cross-reactive effect. To ensure that P140 treatment was leading to a true (non-cross-reactive) mechanism of 'intermolecular tolerance spreading', the goal was thus to demonstrate that T cell response to spliceosomal proteins that do not contain an RNP1 motif was also abolished in P140-treated MRL/lpr mice. U1-C and SmD1 proteins, which are associated to the U1-snRNP particle and are devoid of RNP1 motif, were appropriate candidates in this context. Epitopes recognized by CD4^+ ^T cells are not known in U1-C protein. However, an epitope recognized by T cells from SLE patients and (NZBxNZW)F1 mice has been identified in the C-terminal end of SmD1 [22,23]. This epitope is present in peptide 83–119, which encompasses the sequence 97–119 shown earlier in our laboratory to contain a B-cell epitope recognized by IgG antibodies from MRL/lpr and (NZBxNZW)F1 lupus mice and from patients with lupus [3,10,15]. In preliminary experiments with peptide dose-response measurements, we found that SmD1 peptide 97–119, but not SmD1 peptide 77–96, was efficiently recognized by MRL/lpr PBLs (not shown). With this new target, as above, we analyzed the reactivity of MRL/lpr PBLs (the peptide 77–96 was used as control) after P140 treatment. As shown in Figure 4, we observed that PBLs from P140-treated MRL/lpr mice did no longer proliferate *ex vivo *in response to SmD1 peptide 97–119 used as recall antigen in the culture (81% decrease; *P *= 0.0005).

**Figure 5:**
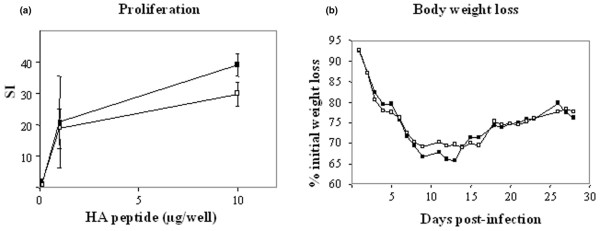
Brief peptide P140 therapy does not affect immune responses to viral challenge in MRL/lpr mice. P140-treated (closed symbols) and untreated MRL/lpr mice (open symbols) (10 mice/group) were challenged intra-nasally with infectious influenza virus. **(a)** Thirteen days after viral challenge, the proliferative response of PBLs to increasing concentrations of HA peptide 307–319 was measured *ex vivo*. Results are expressed as SI. Bars show the mean ± SEM. The average tritiated thymidine incorporation in the absence of peptide and in the presence of Con-A was 50 and 4,000 cpm, respectively. **(b)** Weight loss pattern after intra-nasal viral challenge. The weight of MRL/lpr mice treated or untreated with P140 peptide was compared.

Most importantly, we demonstrated that the tolerogenic effect of P140 peptide on T- and B-cell reactivity to auto-antigens was not generalized to the total T- and B-cell response. We found that P140-treated mice display normal capacity to successfully mount T- and B-cell responses after a viral challenge. This was demonstrated by testing the ability of PBLs from MRL/lpr mice treated with either peptide P140 or PBS alone, and then challenged with an infectious dose of influenza virus, to proliferate (Figure 5a) and secrete IFN-γ (not shown) *ex vivo*, in response to a CD4^+ ^T cell promiscuous influenza hemaglutinin epitope used as recall antigen. As shown, both groups behave very similarly. Both groups also produced similar levels of anti-virus IgG antibodies (not shown) and equally recovered body weight loss resulting from infection (Figure 5b).

## Discussion

A crucial question in our understanding of SLE is to identify the early cellular and molecular events that predispose to the loss of tolerance in individuals and those that trigger the transition from a preclinical status to overt disease and perpetuate the immune-mediated, inflammatory response. The concept of the T-B cell spreading phenomenon based on an initial acute autoimmune response restricted to a few epitopes using a limited number of T-cell clones for expansion is now widely accepted. However, the possible initiator antigens and the mechanism that render this(these) antigen(s) immunogenic in a predisposed context is not clear [1,24-26]. It is also not clear what the link is that drives T-B cell diversification from these putative initial epitopes to a particular set of antigens. Our hypothesis is that the RNP1 motif, which is present in a number of RNA-binding proteins and represents a frequent target for B- and T-cell response, might be central in this process and partly explain autoimmunity to RNP proteins in lupus [12,13].

In this model, the RNP1 motif initiates the spreading of the immune response to the whole protein (intramolecular spreading), then proceeds in an ordered manner to other proteins containing the RNP1 motif, and finally, to proteins that do not contain any RNP1 motif but are colocalized in the same particle (intermolecular spreading). In our previous work [14], we clearly demonstrated that the first step of this process effectively occurs in MRL/lpr lupus mice, in which the immune response diversifies from the RNP1 motif to the whole U1-70K protein (intramolecular spreading). In the present study, we validated the second step of our proposed model [13] by demonstrating that an immune response induced against the RNP1 motif can drive the diversification to other spliceosomal proteins that may or may not contain an RNP1 motif, such as U1A, hnRNP-A2, U1C and SmD1 proteins (intermolecular spreading).

Moreover, we not only confirmed that the sequence 131–151 of the U1-70K protein contains a recurrent epitope recognized by CD4^+ ^T cells from MRL/lpr mice, but we also showed that the RNP1 motif present in other spliceosomal proteins can be recognized by MRL/lpr PBLs. Clearly, the RNP1 sequence ^47^RSLKMRGQAFVIF^59 ^present within the U1-A protein induced a stronger proliferation of MRL/lpr PBLs compared to other RNP1 sequences, which were much less efficient and for some, very poor inducers. It has to be noted that the T cell epitopes of U1-A characterized previously by others [27-29] in MRL/lpr and (NZBxNZW)F1 mice and patients with SLE or mixed-connective tissue disease did not or only partially encompass the RNP1 motif. Epitopes recognized by autoreactive CD4^+ ^T cells either in patients or in murine models of lupus or rheumatoid arthritis are not known in the case of the hnRNP-A2 protein.

The RNP1 motif is present in B-cell epitopes recognized in different proteins by antibodies from autoimmune mice and patients [[13]; and references therein]. In the present study, we show that antibodies elicited after immunizing a rabbit with the peptide 131–151 of the U1-70K protein cross-react with the short RNP1 motif contained within the immunizing sequence but significantly less well with the RNP1 motifs of U1-A and hnRNP-A2 proteins, indicating that in solution these short peptides do adopt different conformations. In Western immunoblotting, antibodies from this immunized outbred rabbit reacted strongly with the cognate protein U1-70K and with U1-A, but also with U1-C and SmD1, which are both devoid of RNP1 motif. These results fit well with the previous findings from several groups, which showed similar intermolecular antibody diversification in longitudinal studies of lupus patients and mice [1,30,31]. However, in our rabbits immunized with the 131–151 peptide, we found no antibodies to dsDNA or chromatin, no antibodies to recombinant SmBB', and no clinical sign of autoimmunity. Immunization of experimental rabbits and mouse models with the proline-rich peptide PPPGMRPP, another key sequence, which is present in several spliceosomal proteins and was reported to trigger spreading in immunized animals [32,33], gave contradictory results in independent studies regarding the presence or not of anti-DNA antibodies and signs of lupus-like autoimmunity [34-36]. To our knowledge, PPPGMRPP peptide administrated in lupus mice was never shown to possess protective or tolerogenic properties.

The reasons that underlie the successful treatment of MRL/lpr mice with peptide P140, which significantly retards the emergence of dsDNA IgG antibodies and the progression of nephritis and prolongs the survival of treated mice, are not yet fully elucidated. We showed here that P140 administration not only abolishes T cell intramolecular spreading to other regions of the cognate U1-70K protein, but also leads to an impressive unresponsiveness of PBLs reacting with RNP1-peptides or longer peptides containing the motif, and with a peptide from the C terminus of SmD1 protein, newly characterized in this study and originated from a spliceosomal protein that does not contain any RNP1 motif. Thus, in treated animals, P140 peptide elicits clearly a state of bystander suppression leading to the downregulation of autoreactive T- and B-cell response to other self-antigens (so-called tolerance spreading [37]). Important questions remain at this stage as to whether P140 peptide induces initial tolerance by playing the role of antagonist or partial agonist of the receptor of autoreactive T cells, or by using another mechanism, as previously discussed [9]. The fact that P140-treated animals could mount a normal immune response to foreign antigens is not an unexpected result if we consider that P140 peptide primarily target the TCR of specific autoreactive T cells.

## Conclusion

**Figure 6:**
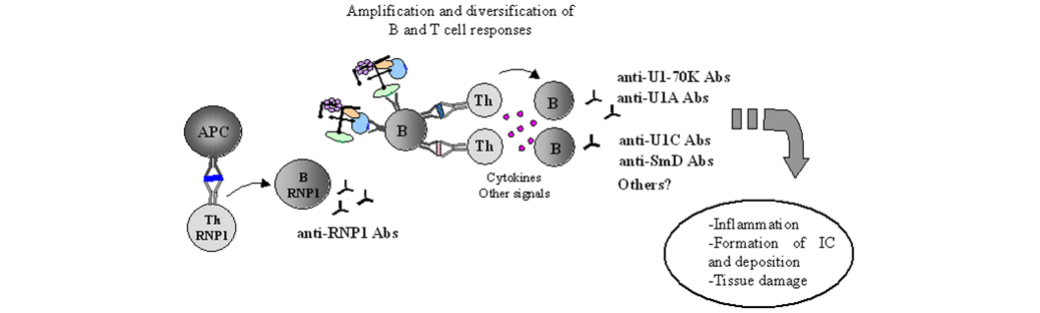
Model illustrating the possible role of RNP1 motif in the initiation of T-B cell spreading pathway (adapted from [13]). Epitopes containing the RNP1 motif (in dark blue) are presented to specific T cells that in turn activate B cells to produce anti-RNP1 antibodies (Abs). These B cells then bind and process the RNP1 epitope present within RNP1+ proteins, such as U1-70K (in blue) and U1-A (in green), but also the whole spliceosomal particle that contains RNP1-proteins, such as U1-C (in yellow) and SmD1 (in pink) proteins. This leads to the activation of Th and B cells and results in the production of diverse sets of auto-antibodies, which then deposit in tissues (IC, immune complexes) and trigger organ damage.

Our results confirm the importance of the RNP1 motif in the pathway of events leading to autoimmunity in lupus. This motif is unique within the 131–151 sequence, which triggers both intramolecular and intermolecular T and B cell diversification. It is contained in peptides recognized by human autoreactive T cell clones [38] and CD4^+ ^T cells from lupus patients [11]. Thus, our experimental data strongly reinforce the hypothetical model we proposed some years ago [12,13] (Figure 6). However, to advance our understanding of this mechanism it will be necessary to elucidate why, in lupus, T and B cells have not been rendered tolerant to the RNP1 motif. Yet, a very positive aspect of our findings is to provide clear evidence that targeting appropriate autoreactive T cells with a single peptide can be sufficient to efficiently immunomodulate the complex autoimmune response in lupus.

## Abbreviations

ELISA = enzyme-linked immunosorbent assay; FA = Freund's adjuvant; PBL = peripheral blood lymphocyte; SLE = systemic lupus erythematosus;

## Competing interests

Several patents (holders CNRS and ImmuPharma) cover the P140 project. The following authors declare that they have financial competing interest, as holders of stocks and/or options in ImmuPharma: SM and JPB. FM received a salary from ImmuPharma and CNRS for 2 years. The CNRS research lab received bench fees for part of this work from ImmuPharma.

## Authors' contributions

FM performed the experimental work, designed the study and prepared the manuscript. VP performed the experimental work. JP performed peptide synthesis, purification and analysis. SM is the head of laboratory and supervisor of the work, she designed the study and prepared the manuscript. All authors read and approved the final manuscript.
